# IL-6 as an integrative biomarker of residual inflammation and visceral adiposity in psoriasis: a VAI threshold-dependent model

**DOI:** 10.3389/fimmu.2025.1699343

**Published:** 2025-12-15

**Authors:** Eva Klara Merzel Šabović, Tadeja Kraner Šumenjak, Miodrag Janić

**Affiliations:** 1 Dermatovenerology Clinic, University Medical Centre Ljubljana, Ljubljana, Slovenia; 2 Faculty of Medicine, University of Ljubljana, Ljubljana, Slovenia; 3 Faculty of Agriculture and Life Sciences, University of Maribor, Hoče, Slovenia; 4 Clinical Department of Endocrinology, Diabetes and Metabolic Diseases, University Medical Centre Ljubljana, Ljubljana, Slovenia

**Keywords:** interleukin-6, psoriasis, visceral adiposity index, systemic inflammation, obesity

## Abstract

**Introduction:**

Psoriasis patients are frequently exposed to residual inflammation and visceral obesity, two factors that synergistically increase cardiometabolic risk.

**Methods:**

We evaluated IL-6 as a potential integrative biomarker linking these pathways in a cross-sectional study including 80 patients with well-controlled skin disease and 20 matched healthy controls. Serum IL-6 was measured by ELISA, and visceral adiposity estimated using the Visceral Adiposity Index (VAI).

**Results:**

Psoriasis patients displayed significantly higher IL-6 than controls (38.1 pg/mL [35.5–41.3] vs. 21.4 pg/mL [19.5–33.4]; p<0.001). A distinct VAI threshold of 1.3 was identified, above which IL-6 levels rose steeply until VAI 2.2 and then plateaued. Patients with VAI ≥1.3 had markedly higher IL-6 and pro-inflammatory cytokines than those below this cutoff. Random forest regression confirmed IFN-γ, IL-1β, IL-12p70, and IL-17 as dominant predictors of IL-6, while HbA1c, FIB-4, and treatment contributed minimally.

**Discussion:**

These findings suggest that IL-6 elevation in psoriasis primarily reflects cytokine-driven residual inflammation, with non-linear amplification once visceral adiposity exceeds a critical threshold. The threshold-dependent IL-6 dynamic highlights a clinically meaningful inflection point, integrating residual inflammation and visceral fat dysfunction, and may guide early cardiometabolic risk stratification and intervention. Prospective validation is warranted.

## 1 Introduction

Psoriasis is a chronic, immune-mediated inflammatory skin disease affecting 2–3% of the global population (1). Beyond cutaneous manifestations, it is increasingly recognized as a systemic condition, frequently associated with metabolic comorbidities such as obesity, metabolic syndrome, insulin resistance, and cardiovascular disease (1, 2). These comorbidities share overlapping inflammatory pathways with psoriatic skin disease, involving cytokines such as tumor necrosis factor (TNF), IL-17, IL-23, and IL-6 (1, 3, 4). Among these, IL-6 plays a central role in bridging innate and adaptive immunity and mediates both psoriatic inflammation and metabolic dysregulation (5). Elevated IL-6 levels correlate with visceral adiposity, insulin resistance, and increased cardiovascular risk across various conditions (6). However, its role in well-controlled psoriasis, particularly regarding residual inflammation and visceral obesity, remains insufficiently explored.

Even when systemic therapies improve or resolve psoriatic skin symptoms, low-grade systemic inflammation often persists, potentially sustaining elevated metabolic risk (7). The impact of this risk is modulated by individual variability in visceral adiposity and its metabolic activity, which may be amplified by persistent circulating inflammatory cytokines (7). The Visceral Adiposity Index (VAI), integrating anthropometric and biochemical parameters, provides a practical measure of both visceral fat quantity and function (8, 9). The VAI thresholds that delineate the onset of metabolic and cardiovascular risks and thus distinguish “metabolically neutral” from “metabolically active” visceral obesity have not yet been established in psoriasis. However, in an adult Sicilian population, Amato et al. identified VAI cut-off values predictive of metabolic syndrome, set at > 1.32 for men and > 1.21 for women, highlighting these thresholds as clinically relevant markers for the early detection of visceral adipose dysfunction (10).

Despite growing awareness of inflammatory-metabolic risk in psoriasis, no widely accepted biomarker integrates residual inflammatory activity with metabolic burden. Such a biomarker could improve risk stratification and guide targeted interventions. Moreover, identifying the point at which visceral adiposity becomes pathologic—consistent with the concept of “adiposopathy”—has important clinical relevance. In this study, we measured serum IL-6 in well-treated psoriasis patients and healthy controls, examining its relationship with key psoriatic cytokines and visceral adiposity assessed by VAI. We aimed to evaluate IL-6 as a unified biomarker of residual inflammation and metabolic risk, and to explore potential threshold effects of visceral adiposity.

## 2 Methods

### 2.1 Study population and design

This cross-sectional study included 80 patients with psoriasis (54 men, 26 women) and 20 age-matched healthy controls. All participants were recruited consecutively from the Dermatology Outpatient Clinic at the Department of Dermatovenerology, University Medical Centre Ljubljana, Slovenia.

Psoriasis patients had been receiving stable and effective treatment for at least six months, either with topical therapy (n = 21) or systemic therapy, including methotrexate (n = 11), adalimumab (n = 14), secukinumab (n = 14), or guselkumab (n = 20). Treatment efficacy was assessed using the Psoriasis Area and Severity Index (PASI). Overall, 87.5% of patients achieved excellent disease control (PASI< 3), including 46% who reached complete skin clearance (PASI = 0), and 12.5% who showed good response (PASI 5–7). Patient baseline characteristics are summarized in **Table 1**.

**Table 1:** Characteristics of patients. Data are presented as the median (interquartile range), or number of cases for categorical variable.

Patients’ characteristics		TOP (n=21)	MTX (n=11)	ADA (n=14)	SEC (n=14)	GUS (n=20)	CG (n=20)
Average age (years)		38.00 (32.00-41.50)	39.00 (35.00-42.00)	39.50 (36.75-41.00)	39.50 (34.50-43.25)	40.00 (36.00-43.00)	34.50 (31.25-39.75)
Sex	Male	13	7	10	11	12	13
	Female	8	4	4	3	8	7
BMI (kg/m^2^)		23.36 (22.59-26.18)	28.05 (22.65-37.51)	27.04 (23.62-30.66)	31.04 (26.75-35.39)	27.50 (24.54-34.96)	24.30 (23.40-26.75)
Waist circumference (cm)		86.00 (77.50-94.50)	104.00 (92.00-121.50)	93.25 (89.125-109.375)	104.50 (98.00-113.50)	99.25 (85.625-108.875)	90.00 (79.75-94.00)
Duration of psoriasis (years)		8.0 (4.5-20.0)	10.0 (5.0-12.0)	20.0 (11.5-25.0)	16.5 (13.75-23.25)	20.0 (15.0-23.5)	/
Duration of treatment (months)		77.0 (47.0-239.0)	29.0 (21.0-47.0)	95.0 (61.0-117.5)	48.0 (33.5-59.0)	31.0 (27.3-51.0)	/
PASI		0.2 (0.1-1.75)	1.2 (0.7-3.2)	0.0 (0.0-0.2)	0.0 (0.0-0.65)	0.0 (0.0-0.6)	/
BSA (m^2^)		1.0 (1.0-1.5)	2.0 (1.0-4.0)	0.0 (0.0-0.25)	0.0 (0.0-1.0)	0.0 (0.0-1.0)	/

TOP, topical therapy; MTX, methotrexate; ADA, adalimumab; SEC, secukinumab; GUS, guselkumab; CG, control group; PASI, Psoriasis Area and Severity Index, BSA, Body Surface Area, BMI, body mass index; VAI, visceral adiposity index.

Inclusion criteria were confirmed diagnosis of psoriasis, age between 30 and 45 years, stable treatment regimen, and satisfactory disease control without planned changes. Exclusion criteria included: previous cardiovascular events, type 1 or type 2 diabetes, menopause, pregnancy or breastfeeding, psoriatic arthritis, other chronic inflammatory conditions, or concurrent non-psoriasis medications.

All participants provided written informed consent. The study was approved by the Slovenian National Medical Ethics Committee (approval no. 0120-422/2021/6), conducted in line with the Declaration of Helsinki (1975, revised 2013), and registered on ClinicalTrials.gov (Identifier: NCT05957120). The study follows the STROBE guidelines (11).

### 2.2 Study protocol

At the study visit, participants underwent a detailed medical history and physical examination. Anthropometric data (weight, height, waist circumference) were recorded, and body mass index (BMI) was calculated. Fasting blood samples were collected using standard venipuncture techniques from the antecubital vein.

### 2.3 Laboratory methods and calculation of metabolic indices

Laboratory samples were processed according to standardized procedures. Biochemical parameters (high-density lipoprotein (HDL) cholesterol, triglycerides), IL-6 and other inflammatory cytokines (TNF, IFN-γ, IL-1β, IL-12p70, IL-17 and IL-23) were determined, as extensively described previously (12, 13).

Metabolic index (visceral adiposity index (VAI)) was calculated using the equation $VAI=\;\left(\frac{WC}{36.58+\left(1.89\;x\;BMI\right)}\right)\;\times \;\left(\frac{TG}{0.81}\right)\;\times \;\left(\frac{1.52}{HDL}\right)\;$ for women and $VAI=\;\left(\frac{WC}{39.68+\left(1.88\;x\;BMI\right)}\right)\;\times \;\left(\frac{TG}{1.03}\right)\;\times \;\left(\frac{1.31}{HDL}\right)$ for men (10).

### 2.4 Statistical analysis

The statistical analysis was performed using R software. As the assumptions for parametric tests were not met, non-parametric methods were applied. The Mann–Whitney U test and the Kruskal–Wallis test were used to compare medians between independent groups, with *post hoc* Dunn’s test and Bonferroni correction for multiple comparisons. Medians and interquartile ranges (IQRs) were reported for descriptive statistics. Spearman’s rank correlation was conducted to assess relationships between variables. To identify the main determinants of circulating IL-6 levels, we used a random forest regression model with 500 trees. This machine learning approach is well suited for complex biological data, as it captures non-linear relationships, handles missing values, and minimizes overfitting. Variable importance was evaluated using the percentage increase in mean squared error (%IncMSE) following permutation. A non-parametric LOESS model was used to investigate the relationship between VAI and IL-6.

## 3 Results

**Figure 1:**
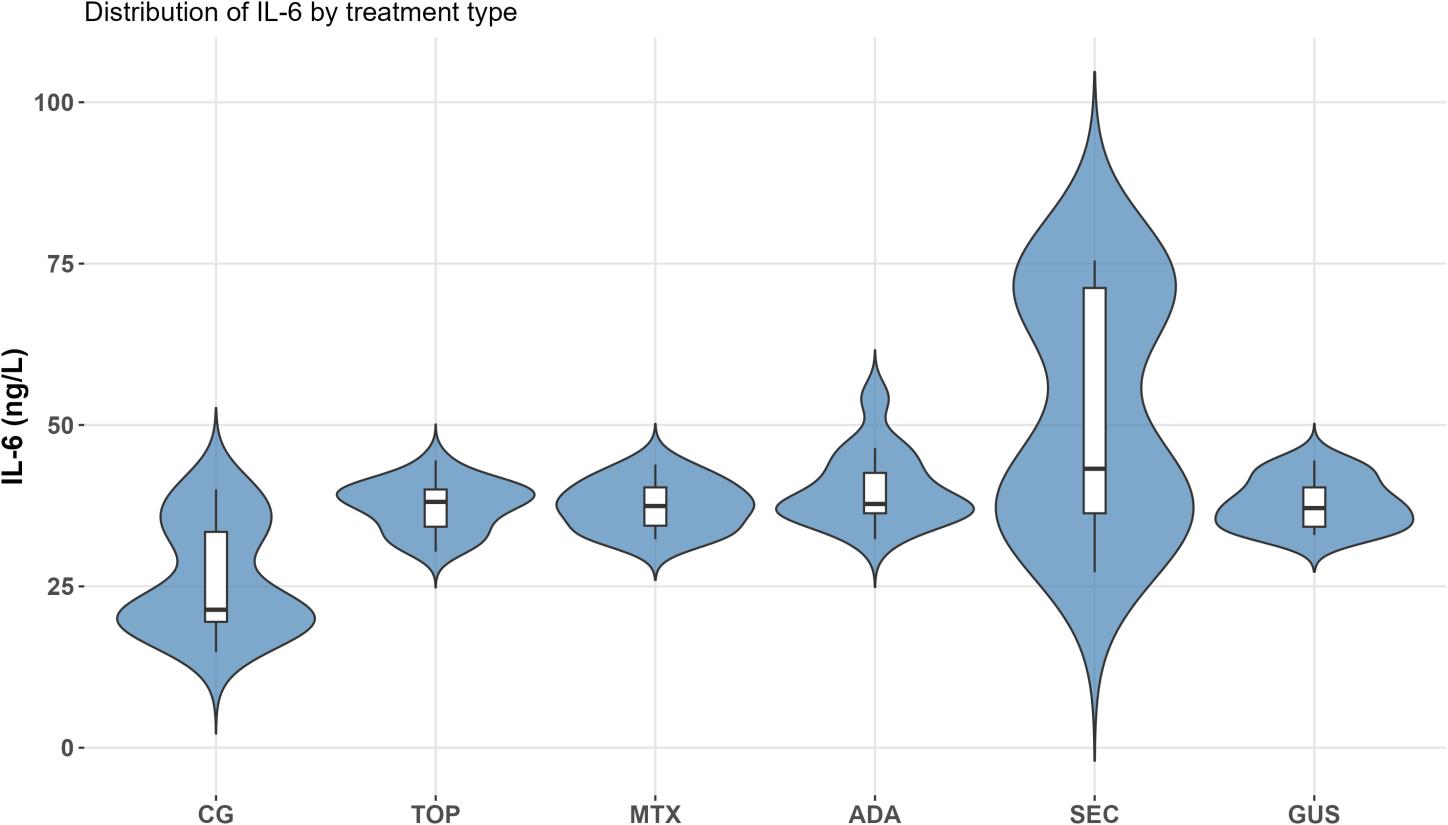
Distribution of IL-6 levels in psoriasis patients across different treatment groups. Violin plots depict the density and spread of IL-6 values. CG, control group; TOP, topical therapy; MTX, methotrexate; ADA, adalimumab; SEC, secukinumab; GUS, guselkumab.

IL-6 levels were significantly higher in psoriasis patients compared to healthy controls (median 38.1 vs. 21.4; *p* < 0.001). IL-6 levels were significantly elevated compared to controls in all groups, except for the methotrexate-treated group, where the increase was near significance (*p* = 0.063). No significant differences in IL-6 concentrations were found between treatment groups: median IL-6 values were 38.1 (topical therapy), 37.4 (methotrexate), 37.8 (adalimumab), 43.2 (secukinumab), and 37.1 (guselkumab). **Figure 1** presents the distribution of IL-6 levels across groups via violin plot visualization.

Patients with a VAI ≥ 1.3 (n=52) had higher IL-6 levels than those with VAI<1.3 (*n* =28) (39.0 vs. 37.1; *p* = 0.117). The levels of inflammatory cytokines - TNF, interferon-γ (IFN-γ), IL-1β, IL-12p70, IL-17, and IL-23 – were also higher in patients with VAI ≥ 1.3 compared to those with VAI< 1.3 (TNF: 48.7 vs 46.4; p=0.057; IFN-γ: 160.2 vs 138.8; p<0.01; IL-1β: 66.6 vs 59.7; p<0.01; IL-12p70: 298.7 vs 271.6; p<0.01; IL-17: 27.4 vs 26.0; p<0.05; IL-23: 1.5 vs 1.4; p=0.118).

**Figure 2:**
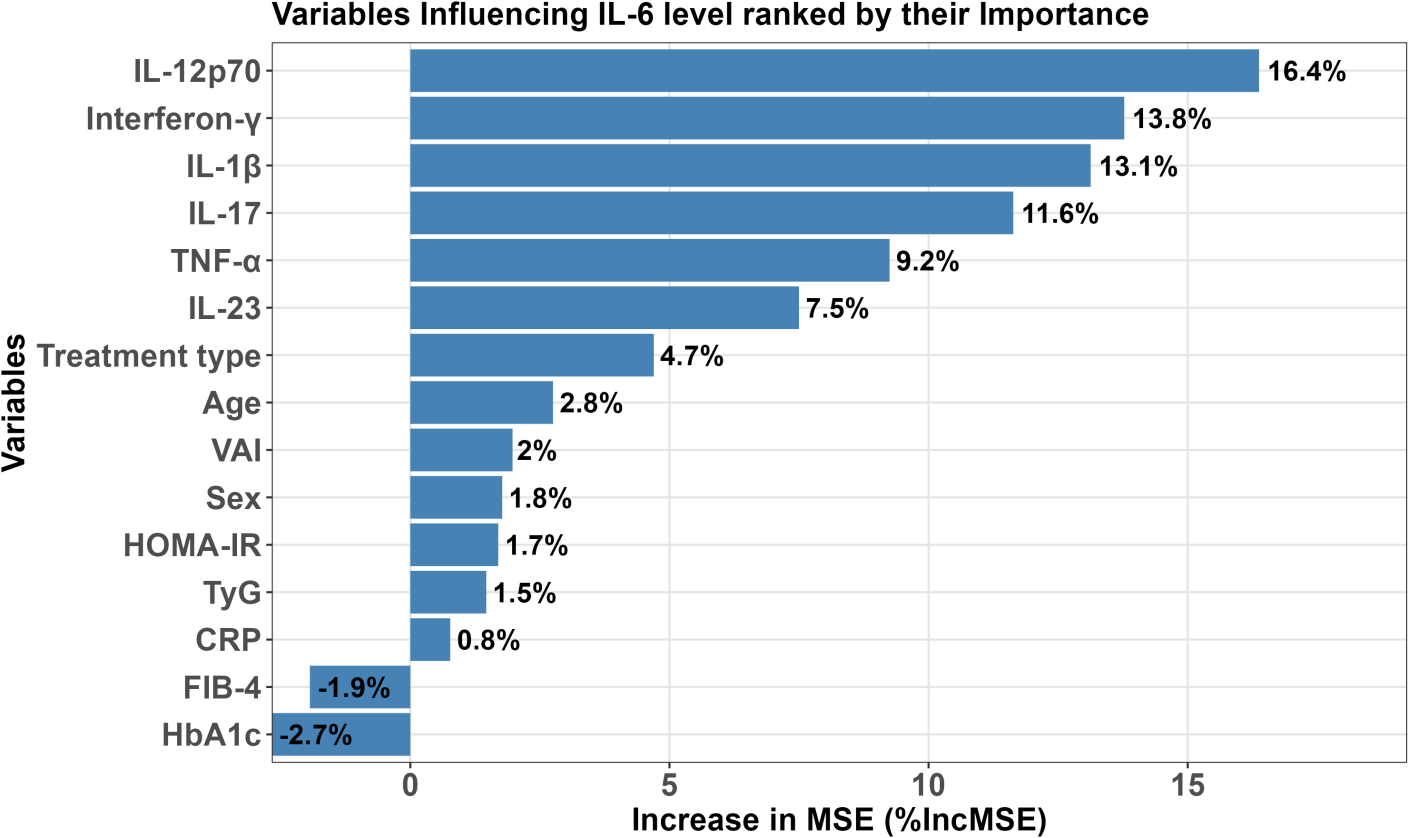
Random forest analysis of predictors of IL-6 levels in psoriasis patients. IL, interleukin; TNF-α, tumor necrosis factor-α; VAI, visceral adiposity index; HOMA-IR, homeostatic model assessment for insulin resistance; TyG, triglyceride-glucose index; CRP, C-reactive protein; FIB-4, Fibrosis-4 Index; HbA1c, glycated hemoglobin.

Random forest regression identified key pro-inflammatory cytokines as the strongest predictors of IL-6 levels: IL-12p70 (%IncMSE = 16.4), IFN-γ (13.8), IL-1β (13.1), and IL-17 (11.6) all demonstrated high importance. TNF (9.2) and IL-23 (7.5) showed moderate contributions. Treatment type (%IncMSE = 4.7). In contrast, metabolic parameters such as HbA1c (–2.7), VAI (2.0), and FIB-4 (–1.9) had minimal or negative contributions to model performance. These findings are summarized in **Figure 2**.

**Figure 3:**
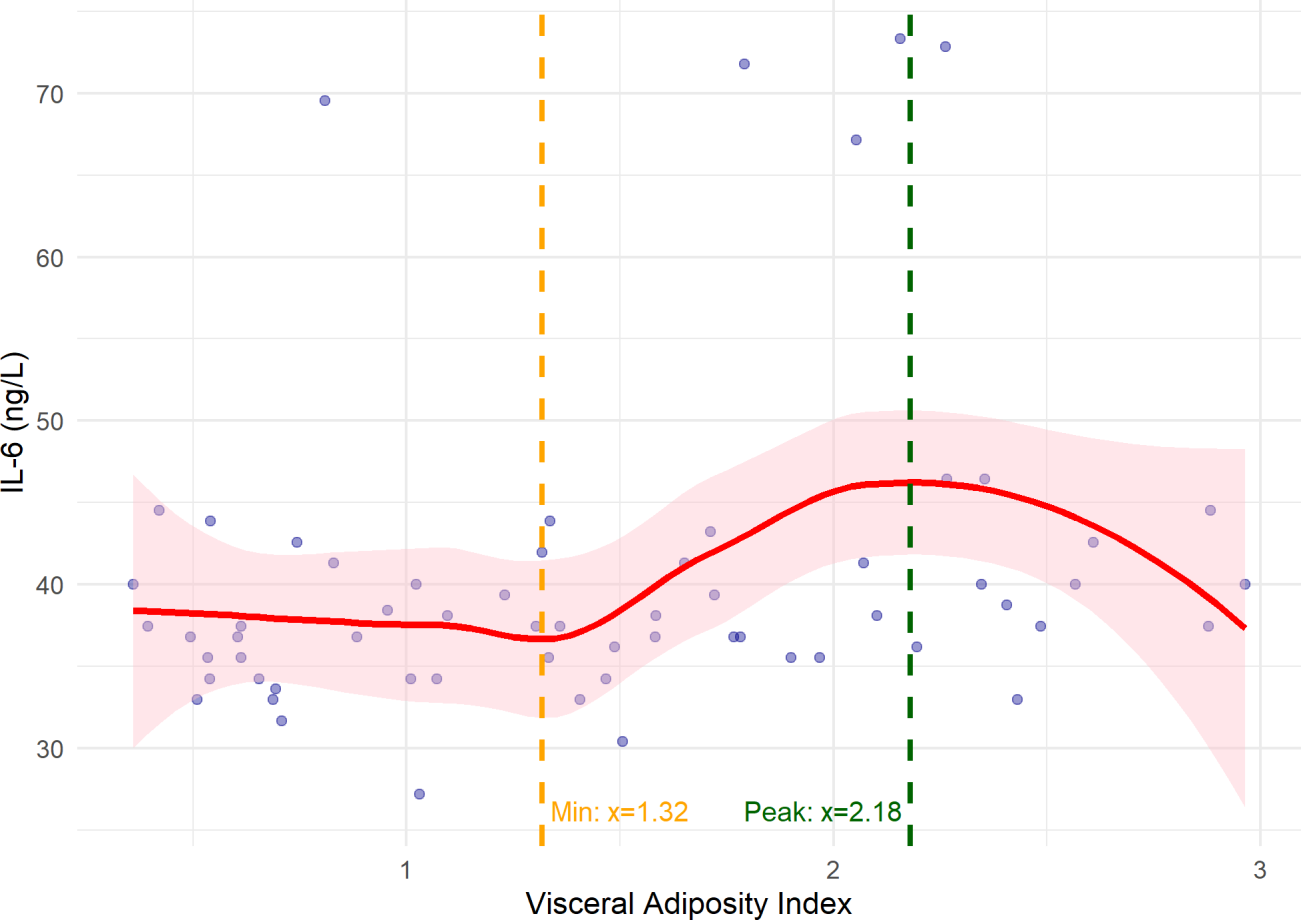
IL-6 levels according to the Visceral Adiposity Index (VAI) in psoriasis patients. IL-6 increased sharply above a VAI of 1.3, rose until approximately 2.2, and then reached a plateau.

The LOESS curve showed a nonlinear trend, with a local minimum at VAI≈1.3. A significant positive monotonic trend was observed between this minimum and the peak at VAI≈2.2 (Spearman rho = 0.436, p = 0.048), whereas no significant trend was found below VAI = 1.3 or above 2.2. These results indicate that IL-6 increases with VAI from the local minimum to the peak value, after which the relationship stabilizes (**Figure 3**). Overall, IL-6 levels in treated psoriasis patients seem to be predominantly influenced by residual pro-inflammatory activity. While metabolic markers demonstrated limited predictive value in the model, visceral adiposity (VAI) was notably associated with elevated IL-6 levels within the VAI range of 1.3 to 2.2.

## 4 Discussion

In this study, we show that IL-6 remains elevated in patients with psoriasis despite achieving near-complete skin clearance, independent of treatment modality (topical agents, methotrexate, or biologics). IL-6 levels were comparable across therapies, closely correlated with residual proinflammatory cytokines, and markedly increased in patients with elevated visceral adiposity, defined by a VAI within the interval 1.3-2.2, above which IL-6 levels plateaued. These results suggest that IL-6 reflects both persistent systemic inflammation and visceral fat dysfunction, contributing to heightened cardiovascular risk and supporting its role as a clinically informative biomarker in well-treated psoriasis.

Our results demonstrate that residual inflammation persists in patients with well-controlled psoriasis, regardless of therapy. While IL-6 is known to be closely linked to multiple proinflammatory cytokine pathways (14–16), our study demonstrates that this association persists in residual low-grade systemic inflammation, even in the absence of overt skin disease. Unresolved inflammatory burden contributes to heightened cardiometabolic risk including insulin resistance, atherosclerosis, and cardiovascular events (14, 17, 18), and continues to pose a critical clinical challenge. These findings highlight the urgent need for biomarkers capable of capturing persistent inflammation, with IL-6 emerging as a particularly promising candidate whose role in well-treated psoriasis has not yet been fully recognized.

We further demonstrate that visceral obesity amplifies residual inflammation through increased cytokine activity, including IL-6. Although visceral adiposity is recognized as a key driver of inflammation in psoriasis (19–22), our data suggest that it is the progression to dysfunctional visceral fat, characterized by pathological endocrine and immune activity, that exacerbates this effect. Importantly, we identified a clinically relevant threshold for dysfunctional visceral adiposity: VAI ≥ 1.3, above which IL-6 levels rise sharply, with the steepest increase observed between VAI 1.3 and 2.2, followed by a plateau beyond 2.2. To our knowledge, this is the first report establishing a VAI threshold for detecting metabolically active, dysfunctional visceral obesity in psoriasis, providing a practical tool for identifying high-risk patients who may benefit from closer cardiometabolic monitoring and early intervention.

Building on these findings, the observed threshold-dependent rise and subsequent plateau of IL-6 within the VAI range 1.3–2.2 is particularly interesting and novel. We hypothesize that below the lower VAI threshold (~1.3), IL-6 primarily reflects residual immune activity, whereas increases in visceral adiposity may amplify pro-inflammatory signaling via hypertrophic and dysfunctional adipocytes, contributing to a steep, non-linear rise that integrates metabolic stress and immune activation. Above the upper threshold (~2.2), IL-6 appears to plateau, possibly due to saturation of cytokine production or regulatory feedback, suggesting that patients in this range have already reached a high systemic inflammatory burden. Similar threshold- or plateau-dependent dynamics are observed in other medical contexts: for example, glycated hemoglobin (HbA1c) rises steeply in micro- and macrovascular risk up to ~7–8 % (23), serum creatinine increases sharply with early declines in glomerular filtration rate (GFR) but shows smaller, non-linear changes with further reductions (24), and N-terminal pro-B-type natriuretic peptide (NT-proBNP) rises with worsening heart failure before plateauing at severe disease stages (25). These examples highlight that biomarkers integrating multiple pathophysiological signals often behave nonlinearly, consistent with our observations for IL-6 in psoriasis.

Random forest analysis identified proinflammatory cytokines (IL-12p70, IFN-γ, IL-1β, and IL-17) as the dominant predictors of IL-6, whereas metabolic parameters, including VAI, HbA1c, and FIB-4, contributed minimally. This indicates that IL-6 elevation in well-treated psoriasis is primarily cytokine-driven. Notably, although VAI showed low importance in the model overall, our data reveal a threshold effect at VAI≥1.3, above which IL-6 levels rise sharply. This non-linear relationship suggests that visceral adiposity only significantly amplifies residual inflammation when it reaches a metabolically active, dysfunctional state. Identifying patients above this VAI threshold may therefore provide a practical approach for detecting individuals at heightened cardiometabolic risk, despite effective skin disease control.

Our findings emphasize the bidirectional relationship between residual inflammation and visceral obesity in well-treated psoriasis. Chronic low-grade inflammation, extending beyond cutaneous lesions, can promote visceral fat accumulation and dysfunction (19–22, 26, 27). In turn, dysfunctional visceral adipose tissue acts as an active endocrine and immune organ, secreting proinflammatory cytokines such as TNF, IL-1β and IL-6, which perpetuate systemic inflammation and increase cardiometabolic risk (19, 20, 27). IL-6 appears to act both as a biomarker and a mechanistic driver of this persistent inflammatory state. Emerging evidence highlights the therapeutic potential of targeting IL-6 signaling pathways in psoriasis (15, 17, 28), and our findings suggest that IL-6–focused interventions may effectively reduce persistent residual inflammation. While prior studies have documented persistent IL-6 elevation and its cardiovascular implications in psoriasis (29, 30), our study adds novel insight by highlighting the combined effect of visceral adiposity and residual cytokine activity on IL-6 across different treatment modalities.

Current assessment of cardiometabolic risk in psoriasis is limited, as conventional models often overlook disease-specific factors such as persistent low-grade inflammation and visceral obesity (4, 31, 32). Our findings suggest that IL-6 integrates both residual inflammation and visceral adiposity, capturing the pathological processes that drive and sustain cardiovascular risk. In addition, elevated IL-6 has been associated with adverse clinical outcomes, such as insulin resistance, type 2 diabetes, atherosclerosis, and cardiovascular events (33), and in other chronic inflammatory conditions, such as rheumatoid arthritis, systemic lupus erythematosus, and chronic kidney disease, it correlates with endothelial dysfunction, increased arterial stiffness, and progression of subclinical atherosclerosis (14, 15, 33).

Beyond traditional cardiometabolic risk, residual inflammation and visceral obesity may jointly increase the risk of cardiac dysfunction in psoriasis, particularly in younger patients. The risk of heart failure is elevated in psoriasis, with adjusted hazard ratios of 1.22 for mild and 1.53 for severe disease compared with the general population (34). This dual-hit mechanism may promote microvascular dysfunction, myocardial fibrosis, and adverse remodeling, predisposing primarily to heart failure with preserved ejection fraction (35). Within this context, IL-6 emerges as a potential biomarker, integrating residual inflammation and the metabolic burden of visceral adiposity to reflect the risk of early myocardial vulnerability and/or dysfunction. These observations warrant validation in large prospective clinical trials, in line with recent reports highlighting the risk of heart failure in psoriasis and younger populations (36–38).

Although these findings are compelling, several limitations should be noted. The study included a relatively small sample size. The cross-sectional design precludes causal inference, and VAI, used as a surrogate for visceral fat, has not been validated against imaging-based measures as MRI or CT. Nevertheless, its simplicity and accessibility make VAI a practical tool for routine clinical application (39, 40). Despite controlling for known confounders, unmeasured factors such as dietary habits, physical activity, or genetic predispositions could influence the observed associations. Furthermore, the study was conducted in a specific population of well-treated psoriasis patients in Slovenia, which may limit generalizability; replication in diverse cohorts is needed. Finally, while we focused on biomarker relationships and threshold-dependent IL-6 dynamics, we did not assess their impact on clinical outcomes, such as cardiovascular events or mortality. Prospective studies are warranted to confirm IL-6’s predictive value for long-term inflammatory burden, metabolic and cardiovascular outcomes, and to further explore its role in guiding personalized management strategies.

In conclusion, our findings highlight IL-6 as a promising biomarker that integrates residual inflammation and dysfunctional visceral adiposity, two central drivers of metabolic and cardiovascular risk in psoriasis. We identified a VAI interval of 1.3-2.2, readily applicable in routine clinical practice, within IL-6 rises steeply, reflecting metabolically active, dysfunctional visceral fat. Measuring IL-6 and VAI may facilitate early identification of patients at elevated cardiometabolic risk despite effective skin disease control, supporting more comprehensive and individualized therapeutic strategies.

## Data Availability

The raw data supporting the conclusions of this article will be made available by the authors, without undue reservation.
